# Differentiation of bland from neoplastic thrombus of the portal vein in patients with hepatocellular carcinoma: application of susceptibility-weighted MR imaging

**DOI:** 10.1186/1471-2407-14-590

**Published:** 2014-08-15

**Authors:** Chuanming Li, Jiani Hu, Daiquan Zhou, Jun Zhao, Kuansheng Ma, Xuntao Yin, Jian Wang

**Affiliations:** Department of Radiology, Southwest Hospital, Third Military Medical University, 30 Gaotanyan Road, Chongqing, 400038 China; Department of Radiology, Wayne State University, Detroit, MI 48331 USA; Department of General Surgery, Southwest Hospital, Third Military Medical University, 30 Gaotanyan Road, Chongqing, 400038 China

**Keywords:** MRI, Susceptibility-weighted imaging, Thrombosis, Portal vein, Hepatocellular carcinoma

## Abstract

**Background:**

Neoplastic and bland portal vein thrombi (PVT) are both common in patients with hepatocellular carcinoma (HCC). The correct discrimination of them is essential for therapeutic strategies planning and survival predicting. The current study aims to investigate the value of susceptibility-weighted imaging (SWI) in differentiating bland from neoplastic PVT in HCC patients.

**Methods:**

20 HCC patients with bland PVT and 22 HCC patients with neoplastic PVT were imaged with non-contrast SWI at 3.0 Tesla MRI. The signal intensity (SI) of the PVT and HCC lesions in the same patients was compared on SW images. The phase values of the PVT were compared between neoplastic and bland thrombi cohorts. Receiver operator characteristics (ROC) analysis was conducted to evaluate the diagnostic ability of the phase values for neoplastic and bland thrombi discrimination.

**Results:**

20 of 22 neoplastic PVT were judged similar SI and 2 were judged lower SI than their HCC. For 20 bland PVT, 19 were judged lower SI and 1 was judged similar SI as their HCC (*P*<0.001). The average phase values (0.361 ± 0.224) of the bland PVT were significantly higher than those of the neoplastic PVT (−0.328 ± 0.127, *P*<0.001). The AUC for phase values in differentiating bland from neoplastic PVT was 0.989. The best cut-off value was −0.195, which gave a sensitivity of 95% and a specificity of 95.5%.

**Conclusions:**

SW imaging appears to be a promising new method for distinguishing neoplastic from bland PVT. The high sensitivity and specificity suggest its high value in clinical practice.

## Background

Portal vein thrombosis is a form of venous thrombosis affecting the hepatic portal vein, which can lead to portal hypertension and a reduction in the blood supply to the liver. Neoplastic portal vein thrombus is found in 6.5%–44% of patients with hepatocellular carcinoma (HCC). It renders a patient unsuitable for aggressive treatment approaches, such as surgical resection or chemoembolization, due to the unusually high incidence of tumor recurrence [1–3]. Bland thrombus occurs in 4.5%–26% of patients with chronic liver disease and in 42% of patients with HCC. It can be resolved after thrombolytic and anticoagulant therapy [4, 5]. Neoplastic and bland portal vein thrombi discrimination is of great clinical significance for determining the therapeutic approach, predicting survival, and assessing candidates for liver transplantation.

T2*-weighted imaging (T2*WI) is sensitive to ferrihemoglobin and hemosiderin based on the local field inhomogeneity generated by the paramagnetic effect of iron particles. T2*WI has been proven useful in cerebral venous thrombosis detection and evaluation [6]. Susceptibility-weighted imaging (SWI), which exploits the susceptibility differences between tissues as a new type of contrast, is more sensitive to detecting focal field inhomogeneity by adding phase information to the T2* contrast. SWI phase imaging avoids the impact of main magnetic field inhomogeneity through the implementation of a high-pass filter [7]. Three-dimensional (3D) SWI has been proven superior to T2* and other existing magnetic resonance imaging (MRI) techniques for the detection of iron content and hemorrhage in brain [8, 9]. Two-dimensional SWI is a newer approach than 3D SWI, and it is nearly immune to breathing artifacts because it takes advantage of breath-holds. This technique has been successfully applied to the analysis of cirrhotic livers [10]. The value of SW imaging has not, to our knowledge, been studied for characterizing intravascular thrombosis in the liver. The purpose of this study was to investigate the value of SW imaging in distinguishing a bland thrombus from a neoplastic thrombus of the portal vein in patients with HCC.

## Methods

### Subjects

This HIPAA-compliant study was approved by the ethics commission of Southwest Hospital of China and written informed consent was obtained from each patient. From Oct 2011 to Dec 2013, 46 consecutive patients who had pathology-confirmed HCC and portal vein thrombus (PVT) participated in this study. 4 patients were excluded because of the following: a history of hepatic surgery, coexisting bland and neoplastic thrombi, or an unsuccessful examination resulting from body movement and artifacts. Thus, a total of 42 patients (20 men and 22 women, with a mean age of 45.3 years, range of 36–65 years; a mean weight of 71.56 kg, range of 47–98 kg) including 22 neoplastic PVT and 20 bland PVT formed the final study cohort. The PVT was localized in: the main portal trunk in 12 cases, the right branch in 16 cases, the left branch in 10 cases, and a combination of these in 4 cases. PVT of 22 patients were confirmed by surgery, 10 were confirmed by biopsy, and 10 were diagnosed based on other imaging criteria according to the litarature [11–14].

### Computed tomographic imaging

Contrast-enhanced multiphase CT was performed with a multi-detector dual-source CT (Definition, Siemens Healthcare, Forchheim, Germany). The examination consisted of precontrast images and three dynamic phase images acquired 35 s (hepatic arterial phase), 70 s (portal venous phase), and 180 s (delayed phase) following the intravenous administration of 100–120 ml Ultravist 370 (Bayer-Schering, Leverkusen, Germany) at a rate of 3–4 ml/s. The imaging parameters were as follows: 250 mAs, 120 kVp, and 1.2 mm beam collimation with a 0.5 s gantry rotation time. The field of view (FOV) was 35 cm, with a reconstruction thickness and interval of 5 mm.

### Magnetic resonance imaging

MR imaging was performed with a 3.0 T whole body system (Magnetom Trio, Siemens Healthcare, Erlangen, Germany) using a standard 12-channel matrix coil without intravenous contrast enhancement. The following MR pulse sequences were used: transverse T1-weighted 2D gradient echo (GRE) (flip angle 70°, TR/TE 140/2.46 ms), transverse T2-weighted 2D fast spin echo (flip angle 122°, TR/ TE =3700/84 ms) and transverse abdominal 2D SWI (flip angle 20°, TR/TE = 150/10 ms). For all of the patients, the following parameters were used: FOV 280 × 285 mm^2^; matrix 384 × 250; 30 slices; and a slice thickness of 5 mm with a gap of 1 mm. The protocol for SWI was similar to that used in a previous study [10]. Three breath-holds were used, each lasting 16 seconds. The total acquisition time was not longer than 1 minute and 20 seconds, including the break time between the breath holds. SWI postprocessing was done inline and consisted of the following steps: 1) Original images from each channel were passed through a 32 × 32 high pass filter to remove background artifacts; 2) The highpass filtered images from each channel were weighted by the coil sensitivity factor and combined to generate a single complex image; 3) highpass filter corrected phase images were created from the final complex images; 4) a normalized phase mask was calculated from each corrected phased image and multiplied with the magnitude image to produce the final SWI and phase image [7].

### Image analysis

All of the SWI images were evaluated with SPIN software (Signal Processing in NMR, Version 1751, MRI Institute for Biomedical Research, Detroit, MI, USA; http://www.mrimaging.com/category.88.html) by two reviewers who were unaware of the bland or neoplastic nature of the thrombi and who had no access to the other sequences. All SW Images were evaluated qualitatively and then quantitatively.

### Qualitative analysis

For qualitative analysis, the readers were asked to compare the signal intensity (SI) of the portal vein thrombi with those of the HCC on the SW images. They classified the SI of the portal vein thrombi into the following categories: higher, equal to, or lower than the SI of the HCC. The readings were performed separately.

### Quantitative analysis

**Figure 1:**
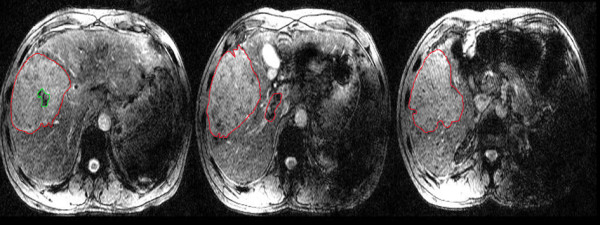
**Example of the ROI over the tumor and thrombus.** Red lines delineate the HCC and PVT, green lines delineate a hemorrhages in the HCC.

Regions of interest (ROI) were drawn directly to delineate the entire HCC and PVT, avoiding any vessels and hemorrhages (Figure 1). The mean and standard deviation (SD) of the Siemens Phase Unit (SPU) were obtained from the entire ROIs and converted into radians using the following equation: (SPU-2048) x π /2048 [15]. The phase values of the tumors in the neoplastic and bland cohorts were compared using the Mann–Whitney test. The phase values of the tumors and thrombi were compared in each group using the Wilcoxon matched pairs signed rank test. The phase values of the thrombi of the two cohorts were compared using the Mann–Whitney test. P values <0.05 were considered statistically significant. Receiver operator characteristics (ROC) analysis was conducted to evaluate the diagnostic ability of phase values for neoplastic and bland thrombi discrimination. The areas under the ROC curve (AUC) and the confidence intervals (CIs) were assessed. The cut-off values that maximized the sum of the sensitivity and specificity were determined and set as the point in the most upper left hand corner. All statistical analyses were performed with the SPSS 17.0 software package (SPSS Inc., Chicago, IL, USA).

## Results

### Qualitative analysis

**Figure 2:**
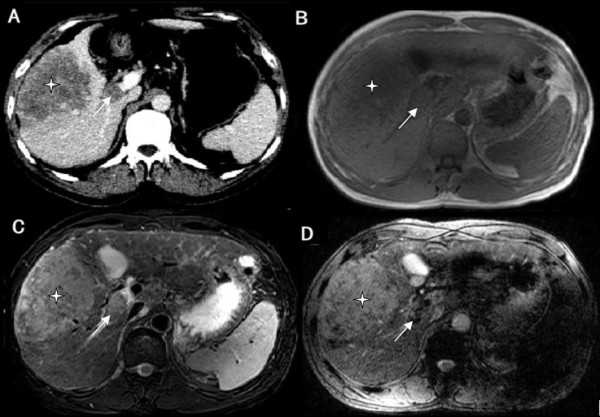
**Bland thrombosis of the portal vein in a patient with hepatocellular carcinoma (HCC). A**: Contrast-enhanced multidetector computer tomography (CE-CT); **B**: T1-weighted imaging (T1WI); **C**: T2-weighted imaging (T2WI); and **D**: Susceptibility-weighted imaging (SWI). HCC (*) is seen occupying the right lobe of the liver. A filling defect is noted in the right portal vein (white arrow), which exhibits lower signal intensity (SI) than the tumor by SWI.

**Figure 3:**
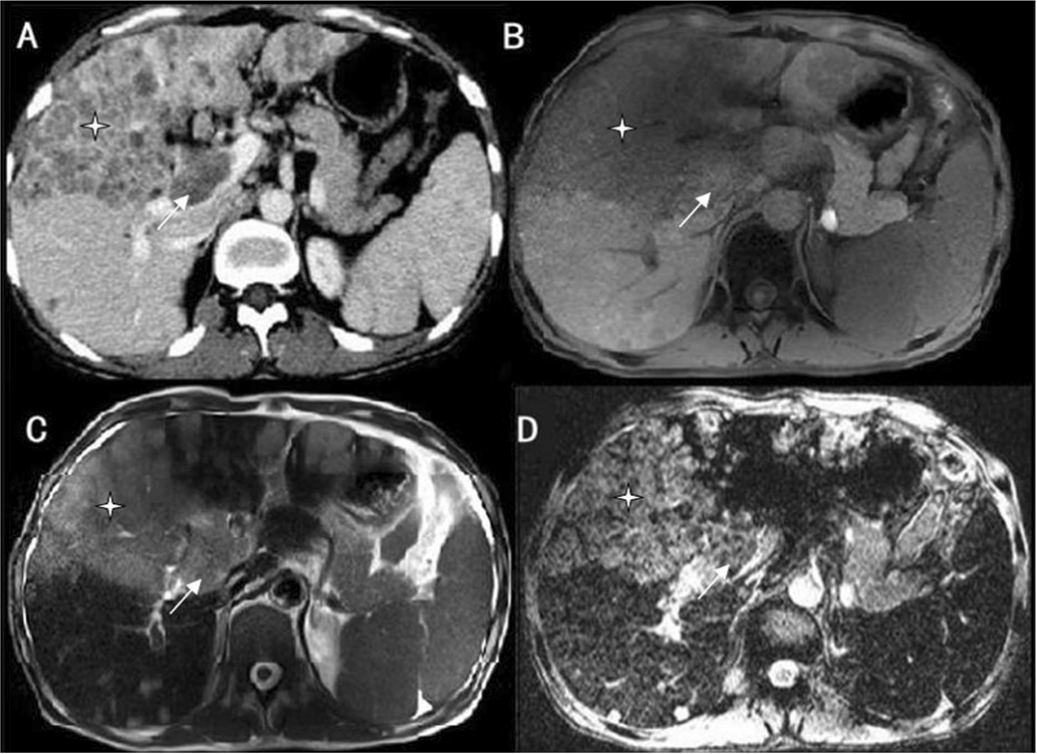
**Neoplastic thrombosis of the portal vein in a patient with hepatocellular carcinoma (HCC). A**: Contrast-enhanced multidetector computer tomography (CE-CT); **B**: T1-weighted imaging (T1WI); **C**: T2-weighted imaging (T2WI); and **D**: Susceptibility-weighted imaging (SWI). A large HCC (*) is seen in the right lobe of the liver and invades the right portal vein (white arrow). The HCC and portal vein thrombus display similar signal intensity (SI) by SW imaging.

20 of 22 neoplastic thrombi were judged similar SI and 2 were judged lower SI than their HCC. For 20 bland thrombi, 19 were judged lower SI and 1 were judged similar SI as their HCC (*P*<0.001) (Figures 2 and 3). There is no significant difference between the two readers (*P*>0.05).

### Quantitative analysis

**Figure 4:**
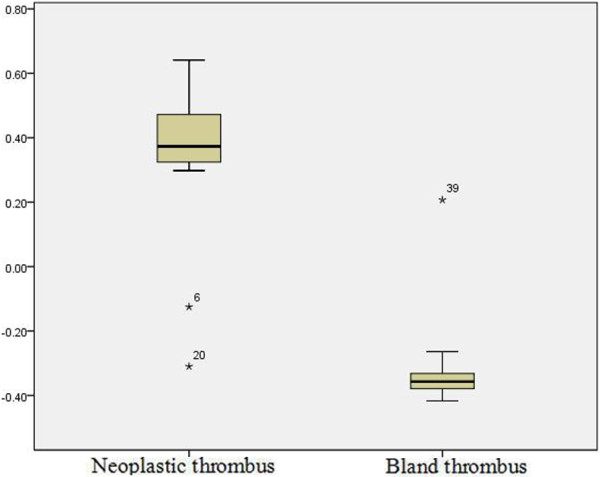
**Phase values (in radians) of neoplastic and bland portal vein thrombi (PVT).**

**Figure 5:**
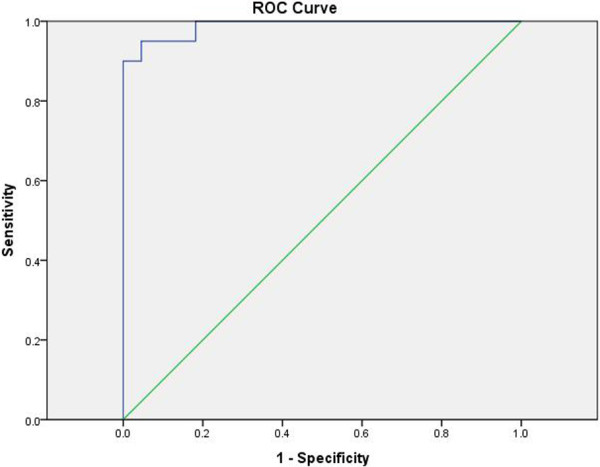
**Receiver operating characteristics curves for phase values (in radians) in neoplastic and bland portal vein thrombi (PVT) discrimination.**

There is a significant phase value difference between bland thrombi (0.361 ± 0.224) and neoplastic thrombi (−0.328 ± 0.127, *P*<0.001). The AUC for phase values in differentiating bland from neoplastic PVT was 0.989. The best cut-off value was −0.195, which gave a sensitivity of 95% and a specificity of 95.5% (Figures 4 and 5).

No statistically significant difference was found between the phase values of the HCC in the neoplastic and bland cohorts (−0.340 ± 0.067, and −0.326 ± 0.049 respectively; *P*>0.05). No statistically significant difference was found between the phase values of the thrombi (−0.328 ± 0.127) and the HCC (−0.340 ± 0.067) in the neoplastic PVT group (*P*>0.05). The phase values of the thrombi (0.361 ± 0.224) were significantly higher than those of the corresponding HCC (−0.326 ± 0.049) in the bland PVT group (*P*<0.001).

## Discussion

The importance of neoplastic and bland portal vein thrombosis discrimination in patients with HCC is well recognized. Bland thrombus develops from sluggish portal blood flow and can be resolved after thrombolytic and anticoagulant therapy. Neoplastic portal vein thrombus is often caused by the direct invasion of HCC and renders a patient unsuitable for aggressive treatment approaches, such as surgical resection, orthotopic liver transplantation, or chemoembolization, due to the unusually high incidence of tumor recurrence. Neoplastic PVT has also been shown to be an important factor in determining the prognosis of patients with HCC. The five-year survival after surgical resection is 12%–39% in patients with neoplastic vascular invasion and 59% in those without [16–18].

Magnetic resonance imaging is of great value in the assessment of PVT [19]. To our knowledge, this is the first study of PVT analysis by SWI. We found that most SWI SI of neoplastic portal vein thrombi were similar to those of the coexisting HCC, whereas the SI of the bland thrombi were generally lower than those of the coexisting HCC. The phase value difference between neoplastic and bland PVT were statistically significant. The best cut-off value of −0.195 (in radians) gave a sensitivity of 95% and a specificity of 95.5%. These results suggest that SWI is a promising tool that can be used for the diagnosis of neoplastic and bland PVT. Quantitative phase shift analysis is better than qualitative SI analysis. Neoplastic and bland thrombi are formed through different pathophysiological mechanisms. Bland thrombus develops from sluggish portal blood flow and is characterized by the presence of fibrin or blood clots without viable cells. SW imaging is an MR technology that has been shown to be sensitive to ferrihemoglobin and hemosiderin, which have only recently been applied in abdominal imaging. SWI does not require intravenous contrast agents or exposure to radiation. This property makes SWI suitable for repeated examinations and follow-up studies, especially for pregnant patients or for patients with a contradiction to contrast media administration.

The reference standard for characterizing portal vein thrombosis is histopathologic examination. However, portal vein thrombus biopsy is an invasive procedure with an associated risk of bleeding [20–23]. Contrast-enhanced ultrasound is notorious for being user dependent, can be difficult in obese patients and is sometimes hampered by the presence of bowel gas [24, 25]. CE-CT is generally accepted as a reliable tool in identifying and characterizing portal vein thrombosis. The imaging criteria for malignant and benign thrombi discrimination using CT are well established. For example, Tublin et al. suggested that the presence of intrathrombus enhancement is highly indicative of neoplastic PVT [26]. However, CE-CT has several disadvantages, including radiation exposure and the use of contrast materials, which can lead to a fatal induction of anaphylaxis and nephropathy [27–29].

This study has several limitations. The main limitation is related to the method we used to generate the ROI for evaluation. In our study, we drew ROIs manually and calculated the phase values over the entire HCC and thrombus, which might introduce unavoidable measurement error. Secondly, due to the cross-sectional group data we could not observe the dynamic SWI in different courses of bland and neoplastic thrombi. Thirdly, because abdomen SWI is sensitive to motion artifacts from respiratory movement, our use of three consecutive breath-hold acquisitions may not be feasible in all cirrhotic patients, especially those with pulmonary compromise from hepatopulmonary syndrome or ascites. Finally, it should be noted, SI of SWI is influenced greatly by sequence parameters, especially echo time. Our results only proved the SI value under current parameters. However, phase shift value is a real, explicable index and will not change with sequence parameters. It is certainly much more reliable.

## Conclusions

Neoplastic and bland PVT are both common in patients with hepatocellular carcinoma. Our results suggest that SW imaging is a promising new method for distinguishing neoplastic from bland macroscopic thrombi. The high sensitivity and specificity suggest its high value in clinical practice.
